# Tricuspid regurgitation in the setting of LVAD support

**DOI:** 10.3389/fcvm.2023.1090150

**Published:** 2023-05-26

**Authors:** Ananya Mitra, Aleem Siddique

**Affiliations:** ^1^ College of Medicine, University of Nebraska Medical Center, Omaha, NE, United States; ^2^ Division of Cardiothoracic Surgery, Department of Surgery, University of Nebraska Medical Center, Omaha, NE, United States

**Keywords:** left ventricular assist device (LVAD), tricuspid regurgitation, heart failure, right ventricular (RV) failure, prognosis, management, tricuspid valve procedures

## Abstract

Tricuspid valve regurgitation (TR) is a common complication of end-stage heart failure. Increased pulmonary venous pressures caused by left ventricular (LV) dysfunction can result in a progressive dilation of the right ventricle and tricuspid valve annulus, resulting in functional TR. Here, we review what is known about TR in the setting of severe LV dysfunction necessitating long-term mechanical support with left ventricular assist devices (LVADs), including the occurrence of significant TR, its pathophysiology, and natural history. We examine the impact of uncorrected TR on LVAD outcomes and the impact of tricuspid valve interventions at the time of LVAD placement, revealing that TR frequently improves after LVAD placement with or without concomitant tricuspid valve intervention such that the benefit of concomitant intervention remains controversial. We summarize the current evidence on which to base medical decisions and provide recommendations for future directions of study to address outstanding questions in the field.

## 1. Introduction

Heart failure with reduced ejection fraction (HFrEF) is a complex syndrome where cardiac output is unable to meet metabolic demands and accommodate venous return; the only curative treatment is cardiac transplantation (1). The relative paucity of organs for transplantation has led to the adoption of left ventricular assist devices (LVADs) to durably support circulation in select individuals. LVADs have proven superior to optimal medical therapy in trials and registry data (2–4). Current best data suggest a 1-year survival rate of more than 80% with LVAD therapy (5).

Functional tricuspid regurgitation (TR) is present to some degree in 88% of patients with HFrEF (6). In patients with significant left ventricular (LV) dysfunction warranting isolated LVAD support, the prevalence rate of severe TR is 11.7% (5). TR is associated with worse outcomes in patients undergoing LVAD implantation—the duration of postoperative inotropic support, hospital stay, and temporary right ventricular assist device (RVAD) requirement are all increased in patients with significant preimplant TR (7). Furthermore, there is a concern over decreased survival rates (7).

An understanding of the pathophysiology, clinical significance, and best management of TR in the setting of LVAD support is necessary, given the prevalence and impact of TR in this population, and this is the focus of this review.

## 2. Pathophysiology of functional tricuspid regurgitation

There is a close relationship between TR and left and right ventricular (RV) dysfunction. In patients under consideration for LVAD therapy, the underlying cardiomyopathy results in severe LV dysfunction. Chronic volume and pressure overload of the left heart leads to cardiac remodeling with ventricular dilation and hypertrophy.

The increased left-sided pressure results in WHO group 2 pulmonary hypertension (PH) and transmission of the hydrostatic pressure to the RV via the pulmonary vasculature. Functional TR is thus strongly linked to the severity of PH (8). The increased afterload causes RV geometric changes (9). In addition, the underlying cardiomyopathy may affect the RV muscle directly, causing RV dysfunction and RV pressure/volume overload.

Geometric changes include enlargement of the RV apically, lengthening of the ventricle, annular dilation of the tricuspid valve (TV), and papillary muscle displacement, leading directly to tricuspid regurgitation (10, 11). Annular dilation and annular area have been linked to the severity of TR (8, 12). Frequently, these patients also suffer from chronic atrial fibrillation, which contributes to dilation of the right atrium (RA) and tricuspid annulus (13). The geometric changes in the RV pull the papillary muscles outward, restricting or tethering/tenting the leaflets of the TV (11, 13, 14). Although TV leaflet tethering is most strongly associated with RV size and geometry, LV function is an independent and weaker contributor (15). This contribution of LV dysfunction may be explained by a displacement of the septal RV papillary muscle and apical displacement of the anterior papillary muscle seen with LV dilation (8, 12, 16). Tethering of the TV leaflets is sufficient to induce regurgitation in patients even in the absence of significant annular dilation (15). Both annular dilation alone and isolated papillary muscle displacement have been confirmed to cause TR in a porcine *in vitro* model (17).

**Figure 1:**
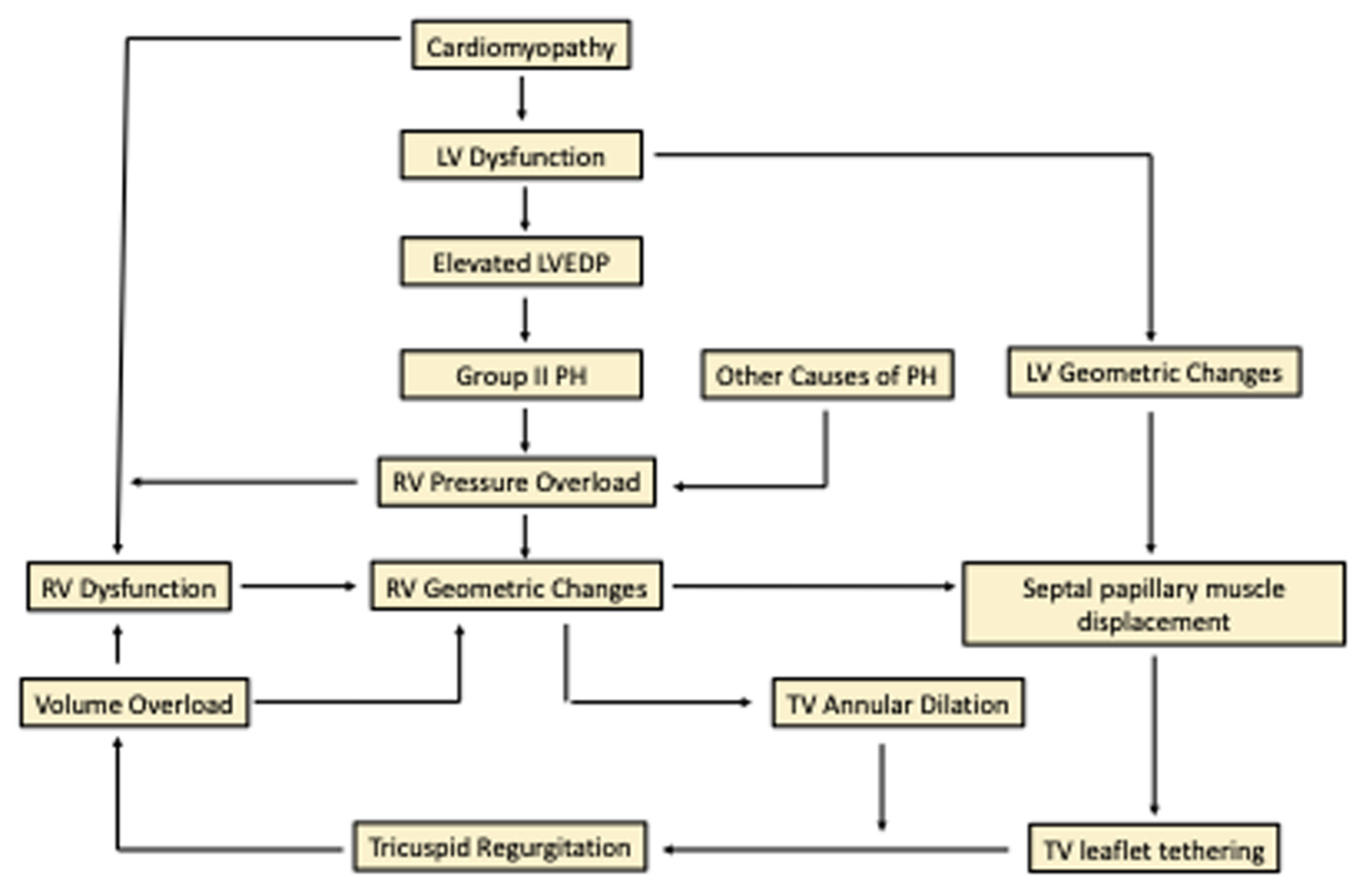
Pathophysiology of tricuspid regurgitation. LV, left ventricle; RV, right ventricle; PH, pulmonary hypertension; TV, tricuspid valve; LVEDP, left ventricular end diastolic pressure.

A positive feedback loop compounds the issue with an increase in volume, worsening geometric changes, and progression of the TR unless the loop is successfully interrupted (Figure 1) (18). Chronic TR results in irreversible cardiac remodeling (19).

## 3. Natural history and impact of uncorrected TR in LVAD placement

RV function is known to be critical to successful LVAD placement (20). As the pathophysiology of TR affects RV function, there are concerns around the clinical impact of significant TR in patients requiring LVAD placement. A review of 2,527 patients in the INTERMACS registry associated the presence of moderate and severe TR pre-LVAD implantation with worse long-term survival (21). Indeed, long-term survival is worse in patients with both preimplant RV dysfunction and preimplant significant TR, suggesting a synergistic detrimental effect (22).

It has been hypothesized that an acute increase in venous return and RV stroke volume from the LVAD can lead to worsening RVF and TR. Conversely, LVAD placement has the potential to interrupt the cycle of volume and pressure overload and improve TR through direct LV unloading.

In examining the effect of LVAD placement in offloading the LV, significant reverse cardiac remodeling was noted within 40 days of implant in hearts explanted at the time of transplant (23). Multiple studies have demonstrated improvements after LVAD placement in pulmonary artery pressures, pulmonary vascular resistance (PVR), pulmonary capillary wedge pressures, and in RV and TV function (22, 24–30). TR improvement after LVAD placement was seen more in patients with a higher PVR, which is likely, as such patients gain from LVAD placement through a substantial decline in PVR (29). Significant echocardiographic improvement in moderate to severe TR occurs in 55%–81% of patients (22, 25, 28, 29). These findings are noted early in the postoperative period, and TR continues to improve over a longer-term follow-up (22, 30). However, not all patients with significant TR show improvement in TV function, and a proportion of patients experience a worsening of TR after LVAD implantation (27, 31).

### 3.1. Effect of preoperative TR on early and late RV function

A study of first-generation LVADs showed that 75% of patients with Grade III or IV TR developed early RVF post-LVAD placement compared with only 12% of patients with grade I or II TR (32). With the continuous flow HeartMate II LVAD, severe preoperative TR was identified as one of several independent predictors for early biventricular support (30). In a randomized trial of LVAD placement with a similar incidence of moderate to severe preoperative TR in both arms (approximately one-quarter), RVF requiring RVAD placement was low and did not vary between the axial flow HeartMate II and the centrifugal flow HeartMate 3 devices (33). In comparison, when looking exclusively at patients with moderate to severe TR undergoing mostly HeartMate 3 placement without TV surgery, the incidence of severe RVF was higher—inotropic support for more than 14 days was needed in 37.5% of patients, and 14.3% of patients required RVAD support (34). Preimplant TR, in combination with elevated RA pressure and end-organ dysfunction, was associated with an increase in early mortality after continuous flow LVAD placement in a large study of the EUROMACS registry (22). These data underline the early hazards related to significant TR.

Late RVF, occurring in 12% of LVAD recipients, is noted to be a frequent cause of death beyond the first year of implant and linked to worse long-term survival (35–37). Preimplantation significant TR was identified as the strongest independent predictor for late RVF; up to 81.2% of patients with late RVF had preimplant moderate or severe TR (38).

### 3.2. Persistent/residual TR after LVAD placement

Critically, patients with residual TR have been identified to have increased long-term mortality, and the persistence of significant TR after LVAD placement is associated with a decline in RV function (22, 29, 31). Table 1 summarizes studies examining the late effects of TR after LVAD placement.

**Table 1:** Summary of studies examining long-term results with significant preoperative TR that is not corrected at LVAD placement.

Author, date, journal	Study groups	Outcomes	Key results	Limitations
Nakanishi et al. (2018). *American Journal of Cardiology* (33)	A total of 274 patients who underwent continuous-flow LVAD placement between 2007 and 2016.	TV annulus and RVF	Greater TV annulus diameter was associated with late RVF with a hazard ratio of 1.221 and diameter measurements of 43.9 vs. 38.2 mm. *p* < 0.001	Retrospective single-center study.
Nakanishi et al. (2018). *Journal of American Heart Association* (31)	A total of 127 patients who underwent isolated LVAD placement between 2007 and 2016.	TV annulus and residual TR after LVAD placement	Greater preoperative TV annulus was associated with increased residual TR. *p* = 0.017	Retrospective single-center study.
		Clinical impact of persistent TR	Residual TR was significantly associated with mortality with a hazard ratio of 5.01. *p* < 0.001	
Gonzalez-Fernandez et al. (2019). *American Journal of Cardiology* (36)	A total of 156 patients who underwent LVAD placement between 2009 and 2018.	Late RVF	A small percentage (10.3) of patients developed late RVF.	Retrospective single-center study.
		Preoperative TR and late RVF	Moderate to severe TR was an independent predictor of late RVF. Hazard ratio 5.50 *p* = 0.02	
Veen et al. (2021). *European Journal of Cardio-Thoracic Surgery* (22)	A total of 2,496 patients who underwent LVAD placement between 2005 and 2018 (EUROMACS registry).	Preoperative TR and 30-day mortality	No significant difference in 30-day morality was seen between mild vs. moderate/severe TR. 10.8% vs. 10.9%. *p* = 0.99	Registry data. Mix of LVADs implanted, limited the ability to determine the impact of a specific device.
		Preoperative TR, RV dysfunction, and long-term survival	The long-term survival rate was lower in patients with moderate/severe TR and RV dysfunction compared with those with good RV function and mild/no TR. 54% vs. 68%	
		Effect of LVAD placement on TR	Moderate/severe TR decreased to mild/none post-LVAD placement in ∼65% patients.	
		Clinical impact of persistent TR	Persistent TR post-LVAD placement was associated with increased mortality with a hazard ratio of 1.16. *p* = 0.001	
Zadok et al. (2022). *Adult Circulatory Support* (29)	A total of 121 patients who underwent LVAD placement between 2009 and 2018.	Effect of LVAD placement on TR	A total of 55% of patients with moderate to severe TR had insignificant TR by 1-year follow-up.	Retrospective single-center study. Some echocardiographic data were missing during the follow-up.
		Clinical impact of persistent TR	Those with persistent TR post-LVAD showed a worsening of RV function, decline in RV work index, and higher loop diuretic use but no significant difference in long-term survival.	

LVAD, left ventricular assist device; RVF, right ventricular failure; TR, tricuspid regurgitation; RV, right ventricle; TV, tricuspid valve.

Several authors have attempted to identify factors that might predict persistent TR after LVAD placement. In one study, residual TR was associated with preoperative TV annulus diameter but not with leaflet tethering (31). Patients with atrial fibrillation are less likely to see an improvement in TR post-LVAD placement, probably because the etiology of their TR includes RA dilation from atrial fibrillation and is less positively impacted by LVAD implantation (29). Atrial fibrillation has also been weakly associated with a progression of TR after LVAD placement (39).

## 4. Impact of concomitant TV surgery at LVAD implant

While significant TR is frequently identified in patients undergoing LVAD placement, the decision to opt for concomitant tricuspid valve intervention (TVI) is controversial. Intervention at the time of LVAD placement could consist of tricuspid valve repair (TVr) or replacement. In practice, repair with an annuloplasty ring has been the dominant mode of TVI (40). Performance of a TVI increases cardiopulmonary bypass (CPB) time and may require cardiac arrest; both of which have the potential to increase operative risk and RVF (26, 41).

Initial experience in a cohort with older-generation LVADs showed a reduction in inotrope use, renal dysfunction, and length of hospital stay in patients of the TVI group as well as a non-significant reduction in the use of RVADs (42). A more recent study of continuous flow LVADs comparing concomitant TVI with isolated LVAD placement in patients with severe TR found a decrease in 30-day readmissions with TVI (43). However, there was no difference in RVF, survival, or TR recurrence.

Two small series identified no substantial difference in outcomes for patients undergoing TVI with LVAD placement and those receiving LVAD implants without TVI, but the groups without TVI did not have significant TR, rendering the comparison difficult (44, 45). Others, including a meta-analysis, found no outcome benefit to TVI, including in clinical measures of RVF or survival (21, 41, 46). A recent propensity-matched cohort of the EUROMACS registry identified patients undergoing TVI to have an intensive care unit (ICU) stay lengthened by 4 days with no benefit in clinical outcomes (26). In this cohort, moderate to severe TR was less prevalent in patients with TVI immediately after surgery but became comparable with time.

A large single-center series with a mix of continuous flow LVADs revealed an improvement in TR with TVI at the expense of increased bleeding and transfusion and no improvement in clinical outcomes (47).

Of concern, TVI was associated with increases in operative time, length of inotropic support, ventilatory support, and ICU stay as well as morbidities such as bleeding, transfusion, RVF, and renal failure in three small single-center series (48–50). In a larger study of patients with moderate to severe TR from the STS database, LVAD placement with concomitant TVI, in comparison with LVAD alone, did not affect RVAD use or death but did increase the risk for renal failure, transfusion, reoperation, ventilator, ICU, and hospital length of stay. Similarly, an analysis of the INTERMACS database associated TVI with increased bleeding, arrhythmia, stroke, and mortality (51).

Methodological concerns in these studies include their retrospective nature, unequal comparator groups particularly with respect to TR severity, and the possibility of selection bias. The TVVAD study randomized patients at a single center with moderate or severe TR to LVAD alone or with concomitant TVI and utilized a primary endpoint of RVF. This study predominantly utilized the current generation of continuous flow LVAD (HeartMate 3, Abbott). Early published results demonstrate an improvement in TR with no substantive clinical benefit, including in the primary endpoint, survival, or adverse events (34). The parameter of quality of life measured by using the Kansas City Cardiomyopathy Questionnaire was also similar between the two groups.

Long-term failure of TVr is an additional concern. In 156 patients with continuous flow LVADs, 37.8% were identified as having a failed TVr defined as moderate or severe TR on any postoperative echocardiographic follow-up (52). Postintervention significant TR (recurrent TR) has been associated with RVF and worse heart failure-free outcomes (46, 52).

Taken together, the data do not currently support TVI at the time of LVAD placement for patients with significant TR. Clinical benefit has not been conclusively demonstrated, and risks such as bleeding, organ dysfunction, and prolongation of various indices of hospital care have been identified and are likely a sequela of prolonging CPB.

Why is TV surgery not helpful for this patient population despite the association of preoperative TR with worse post-LVAD clinical outcomes? There are several hypotheses, and the following are some of them: (1) TR improves in the majority of patients with LVAD therapy such that TVI for all would “overtreat”; (2) TR persists in some patients despite TVI raising the possibility that a different surgical strategy might be more effective in the long-term treatment of TR; (3) TR develops *de novo* in some patients who do not have significant TR at LVAD implant, thus making it hard to draw meaningful comparisons with a control “no pre-operative TR” group; (4) TR is a marker of ventricular dysfunction, does not directly affect clinical outcomes, and thus, interventions aimed at TR do not improve outcomes; and (5) TV surgery involves operative time and risk that negate the benefit.

## 5. *De novo* significant tricuspid regurgitation after LVAD placement

During the follow-up of LVAD recipients, incidence rates range from 6% to 20% of the development of significant TR in patients with none or mild preoperative TR (22, 28, 29, 31). The function of the RV in this subpopulation has not been defined in the available literature and no preoperative clinical/echocardiographic or operative parameters that predict the development of TR after LVAD placement have been identified (28, 29). It is unclear whether this subset of patients with *de novo* TR carries a risk of RVF or worse long-term prognosis compared with patients with insignificant TR or resolved TR after LVAD placement; this is an area for future investigations.

## 6. Future directions

Several outstanding questions related to the natural history and best management of TR in the setting of LVAD therapy remain, which should guide future directions of study (Table 2).

**Table 2:** Future directions for understanding and managing tricuspid regurgitation in the setting of LVAD support.

Future directions
Identify patients with preoperative TR at the greatest risk for early RV dysfunction after LVAD placement and direct such patients toward preoperative optimization and biventricular strategies including cardiac transplantation.
Identify subsets of patients with significant preoperative TR who might benefit from concomitant TV intervention at LVAD placement.
Tailor the TV surgical technique and intervention to the individual TV and RV geometry for achieving best long-term results.
Examine the effect of LVAD settings on TR. Examine independently the impact of future LVAD designs on TR.
Focus on surveillance and management of patients with TR *after* LVAD placement.
Investigate the role of percutaneous TV interventions particularly for patients with TR after LVAD placement.
Identify patient populations at risk of developing *de novo* TR after LVAD placement; understand its natural history and impact.

TR, tricuspid regurgitation; LVAD, left ventricular assist device; TV, tricuspid valve; RV, right ventricle.

It remains unclear whether TR is a marker for RV dysfunction and a predictor of worse clinical outcomes in LVAD recipients or whether it is a causative agent. Longer-term follow-up of the randomized TVVAD trial will be important to clarify the predictors of worse clinical outcomes, the role of TVI, the durability of TVr, and the clinical impact of persistent or recurrent TR.

It is possible that the particular unloading pattern (axial vs. centrifugal flow) of the LVAD implanted affects TR in a way that has not been well defined. In addition, the setting of the LVAD might be impactful with a higher speed unloading the LV more but also, perhaps, increasing venous return. Most published studies include a heterogenous group of LVADs. Future studies of advancing LVAD technology, or studies that include historical devices, separate based upon the type of LVAD based on the LVAD implanted and also to examine the effect of LVAD setting on TR.

Subgroups of patients with significant TR that might benefit from concomitant TVI should be studied. These could include those with TR pathophysiology least likely to respond to isolated LVAD placement. Potential candidates would be patients (1) with severe TR, as most studies to date combine moderate and severe TR, (2) with a dilated TV annulus, (3) with tethered leaflets, and (4) with preoperative atrial fibrillation, as it contributes to the pathophysiology of TR and is associated with persistent TR after LVAD placement. Similarly, if subgroups with the highest early RVF risk are identified, they might be preferred for heart transplantation over LVAD placement.

The current preferred strategy for TVr with an annuloplasty needs re-evaluation. In a non-LVAD setting of TV repair with annuloplasty, TV tethering was the strongest predictor of residual TR (53). Based on TV pathology, certain patients, such as those with significant TV tethering, may warrant a consideration of complex repairs or valve replacement (53, 54). What is the role of percutaneous TVI with edge-to-edge repair in this population? Benefits might lie in avoiding prolongation of the index operation and shifting the focus on patients with TR after LVAD placement.

Patients with TR after LVAD placement could be classified as persistent, recurrent after TVI, or *de novo*. They warrant more attention through heightened surveillance and an understanding of the etiology of their TR, natural history, and best management.

## 7. Conclusion

Significant TR is commonly found in patients with severe LV dysfunction under consideration for LVAD placement. Although its pathophysiology is delineated, and it has been linked to worse clinical outcomes, the best management of significant TR at the time of LVAD placement and afterward remains unclear. TR after LVAD placement is of particular concern as it is linked to progressive RV dysfunction and associated morbidity. These patients warrant further study to understand their best management.
